# Myosin II Synergizes with F-Actin to Promote DNGR-1-Dependent Cross-Presentation of Dead Cell-Associated Antigens

**DOI:** 10.1016/j.celrep.2018.06.038

**Published:** 2018-07-11

**Authors:** Oliver Schulz, Pavel Hanč, Jan P. Böttcher, Robbert Hoogeboom, Sandra S. Diebold, Pavel Tolar, Caetano Reis e Sousa

**Affiliations:** 1 Immunobiology Laboratory, The Francis Crick Institute, 1 Midland Road, London NW1 1AT, UK; 2 Immune Receptor Activation Laboratory, The Francis Crick Institute, 1 Midland Road, London NW1 1AT, UK; 3 Biotherapeutics Division, National Institute for Biological Standards and Control, Potters Bar, Hertfordshire EN6 3QG, UK; 4 Division of Immunology and Inflammation, Imperial College London, Du Cane Road, London SW7 2AZ, UK

**Keywords:** DNGR-1, CLEC9A, C-type lectin, dendritic cells, actin, actin-binding proteins, myosin II

## Abstract

Conventional type 1 DCs (cDC1s) excel at cross-presentation of dead cell-associated antigens partly because they express DNGR-1, a receptor that recognizes exposed actin filaments on dead cells. *In vitro* polymerized F-actin can be used as a synthetic ligand for DNGR-1. However, cellular F-actin is decorated with actin-binding proteins, which could affect DNGR-1 recognition. Here, we demonstrate that myosin II, an F-actin-associated motor protein, greatly potentiates the binding of DNGR-1 to F-actin. Latex beads coated with F-actin and myosin II are taken up by DNGR-1^+^ cDC1s, and antigen associated with those beads is efficiently cross-presented to CD8^+^ T cells. Myosin II-deficient necrotic cells are impaired in their ability to stimulate DNGR-1 or to serve as substrates for cDC1 cross-presentation to CD8^+^ T cells. These results provide insights into the nature of the DNGR-1 ligand and have implications for understanding immune responses to cell-associated antigens and for vaccine design.

## Highlights

• Myosin II amplifies the activity of the DNGR-1 ligand F-actin
• Lack of myosin II in donor cells reduces DNGR-1-dependent cross-presentation
• Beads with F-actin and myosin II can target antigens to cDC1 for CD8 T cell priming

## Graphical Abstract

**Figure:**
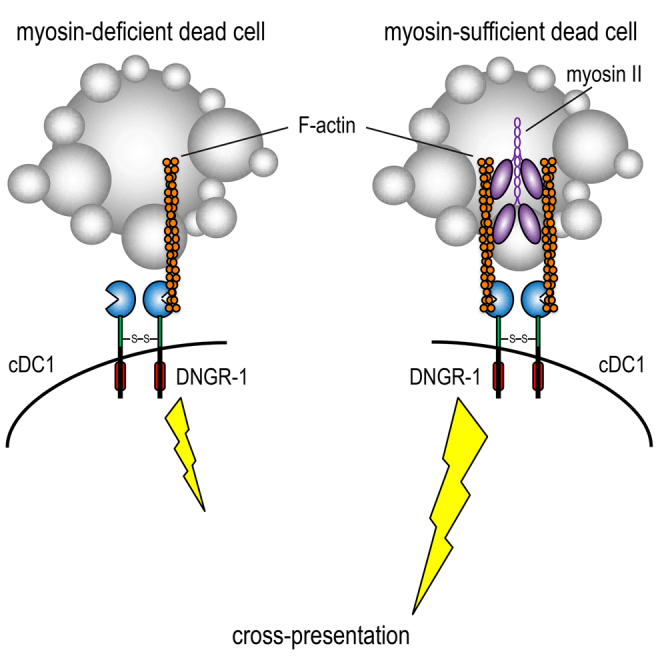

## Teaser

Schulz et al. show that dead cells lacking myosin II have a diminished capacity to serve as antigen donors for dendritic cells that express the DNGR-1 receptor. DNGR-1 binds to F-actin exposed on cell corpses, and myosin II organizes actin filaments into bundles that cross-link the receptor more efficiently.

## Introduction

Cell death resulting from infection or tissue injury can lead to release of damage-associated molecular patterns (DAMPs) (Land, 2003) with immunomodulatory activity (Chen and Nuñez, 2010, Land et al., 1994, Matzinger, 1994, Rock et al., 2011). DAMPs are pre-synthesized cellular molecules such as metabolites (ATP and uric acid), nucleic acids (DNA and RNA), or proteins (heat shock proteins [HSPs] and HMGB1) but can also be considered to include mediators actively produced by the dying cell as a result of death receptor pathways intersecting with nuclear factor κB (NF-κB) signaling (Yatim et al., 2015, Zelenay and Reis e Sousa, 2013). DAMPs are normally sequestered within healthy cells but are released or exposed by dead cells following loss of plasma membrane integrity. Recognition of DAMPs by innate immune receptors, including C-type lectin receptors (CLRs) on myeloid cells, promotes production of pro-inflammatory mediators (Chen and Nuñez, 2010, Rock et al., 2011, Sancho and Reis e Sousa, 2013, Zelenay and Reis e Sousa, 2013). In dendritic cells (DCs), DAMP recognition also plays a role in the extraction of dead cell-associated antigens for presentation to CD8^+^ T cells in a process called cross-presentation (Cruz et al., 2017, Hanč et al., 2016a, Joffre et al., 2012). The most efficient cross-presenting DCs in the mouse, initially identified by the expression of CD8α in lymphoid organs and CD103 in peripheral tissues (Joffre et al., 2012, Shortman and Heath, 2010), have been renamed conventional type 1 DCs (cDC1s) (Guilliams et al., 2014, Schraml and Reis e Sousa, 2015) and require the transcription factors Batf3 (Hildner et al., 2008), ID2 (Hacker et al., 2003), and IRF8 (Aliberti et al., 2003, Schiavoni et al., 2002) for their development. These cells are also found in humans and can be identified across species by expression of the chemokine receptor XCR1 (Dorner et al., 2009), as well as high levels of the CLR DNGR-1 (also known as CLEC9A) (Caminschi et al., 2008, Huysamen et al., 2008, Poulin et al., 2012, Poulin et al., 2010, Sancho et al., 2008). In addition to acting as a cDC1 marker, DNGR-1 is a DAMP receptor. It is expressed as a dimeric transmembrane protein with two C-type lectin-like domains (CTLDs) that face the extracellular space (or the lumen of endosomes) and bind to the cytoskeletal component F-actin, which is exposed in dead cells (Ahrens et al., 2012, Zhang et al., 2012). DNGR-1 binding to F-actin on cell corpses encountered or ingested by cDC1 provokes signaling via Syk and somehow allows endocytic cargo to be shuttled into the cross-presentation pathway (Zelenay et al., 2012). Consistent with a key role for DNGR-1 and cDC1 in cross-presentation of dead cell-associated antigens, loss of DNGR-1 in mice reduces cross-priming of CD8^+^ cytotoxic T cells against model antigens contained within necrotic cells and of viral antigens expressed by cells infected with cytopathic viruses (Iborra et al., 2012, Sancho et al., 2009, Zelenay et al., 2012).

We have solved the structure of DNGR-1 in complex with single-actin filaments using cryo-electron microscopy (Hanč et al., 2015). The structure showed that the CTLDs of DNGR-1 bind to the interface of the two F-actin protofilaments and confirms that naked F-actin is sufficient as a DNGR-1 ligand (Hanč et al., 2015). However, in the context of dead cell recognition, engagement of DNGR-1 could involve an additional factor or factors. This is because F-actin in cells is always coated with actin-binding proteins (ABPs), a group of more than 200 proteins that associate with G- and/or F-actin and regulate its polymerization, generation of contractile force, and actin-dependent cell motility (dos Remedios et al., 2003, Pollard and Cooper, 1986). This prompted us to test purified ABPs for the ability to potentiate or block DNGR-1 binding to F-actin. Here, we report that most ABPs tested did not affect the interaction of DNGR-1 with F-actin, with the exception of the motor protein myosin II. F-actin combined with myosin II bound to DNGR-1 more efficiently than naked F-actin and showed increased agonistic activity. Consistent with that notion, antigen particles bearing F-actin and myosin II were efficiently taken up and cross-presented by DNGR-1^+^ cDC1s, while corpses of cells lacking myosin II were reduced in their ability to stimulate DNGR-1 and to serve as antigen sources for cross-presentation. Our results indicate that F-actin and myosin complexes are the physiological substrates for DNGR-1-dependent recognition of dead cells and can be exploited for the purpose of vaccination.

## Results

### Myosin II Increases the Binding of DNGR-1 to F-Actin

**Figure 1:**
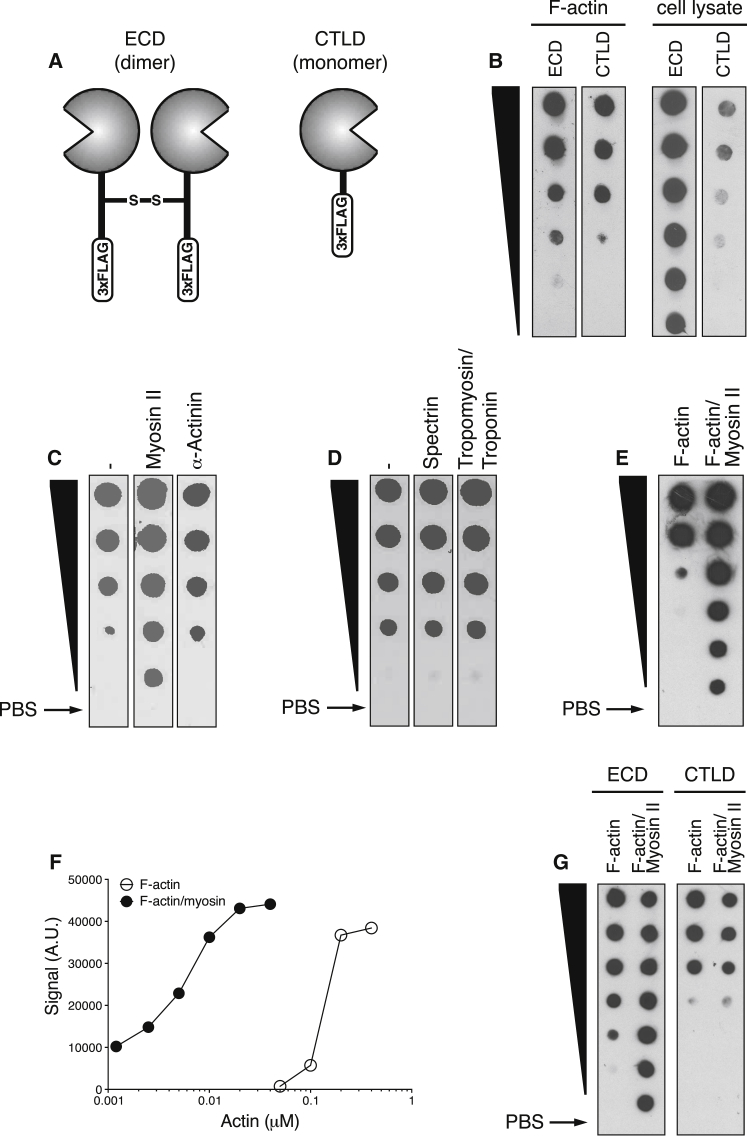
Addition of Myosin II to F-Actin Promotes DNGR-1 Binding Serial (2-fold) dilutions from top to bottom (black wedge) of *in vitro* polymerized F-actin (top concentration: 0.4 μM in E and 0.2 μM in B–D and G) or F-actin complexed with myosin II (top concentration: 0.04 μM in E and 0.2 μM in G) were analyzed by dot blot. Arrows indicate PBS control dots. (A) Schematic representation of soluble DNGR-1 reagents: ECD dimer (left) and CTLD monomer (right). (B) DNGR-1 ECD and DNGR-1 CTLD (20 μg/mL ) binding to immobilized F-actin and HeLa cell lysate. (C and D) Pre-incubation of immobilized F-actin with either blocking buffer (control), myosin II or α-actinin (C) or blocking buffer (control), spectrin or tropomyosin/troponin (D) (all at 10 μg/ml). (E and F) Titration of F-actin and F-actin and myosin II complexes (the latter starts at 10-fold lower concentration) (E) and dose-response curve (F) after quantitation of the signal in (E) using ImageJ software. (G) DNGR-1 ECD and DNGR-1 CTLD (20 μg/mL) binding to immobilized F-actin and F-actin and myosin II. Data are representative of 2 (C, D, and G) and 3 (B, E, and F) independent experiments.

DNGR-1 binding to ligand can be monitored experimentally by using the soluble extracellular domain (ECD) of the DNGR-1 dimeric receptor or the monomeric CTLD (Figure 1A) (Ahrens et al., 2012, Sancho et al., 2009). We compared dimeric ECD and monomeric CTLD for their ability to detect *in vitro* polymerized F-actin or F-actin in cell lysates immobilized onto a nitrocellulose membrane. Although both reagents bound to *in vitro* polymerized F-actin with similar efficiency, the dimeric ECD bound more efficiently to F-actin in cell extracts (Figure 1A). This observation suggested that the cellular ligand for DNGR-1 is not fully mimicked by *in vitro* polymerized F-actin and suggested that DNGR-1 and F-actin interactions are governed by additional cellular factors. Because immunoprecipitation of cell lysates with DNGR-1 ECD led to enrichment not only for actin but also for other cytoskeletal proteins (Ahrens et al., 2012), we tested a variety of cellular ABPs for their ability to modulate binding of DNGR-1 to *in vitro* polymerized F-actin. Most ABPs examined, including α-actinin, spectrin, and tropomyosin and troponin, did not grossly affect binding of DNGR-1 ECD to F-actin (Figures 1C and 1D). However, we noticed increased binding of DNGR-1 ECD to immobilized F-actin pre-treated with myosin II, an actin bundling motor protein (Figure 1C). Titration of F-actin or pre-assembled F-actin and myosin II complexes on a dot blot revealed that myosin II improved DNGR-1 ECD binding by at least 50-fold (Figures 1E and 1F). Enhanced binding to F-actin and myosin II was not seen with the DNGR-1 CTLD despite the ability of the latter to bind naked F-actin as efficiently as DNGR-1 ECD (Figure 1G). This observation suggests that myosin II facilitates co-operative binding of the two CTLDs in the DNGR-1 dimer to the F-actin ligand.

### Intact Myosin II Potentiates the Agonistic Function of F-Actin for DNGR-1

**Figure 2:**
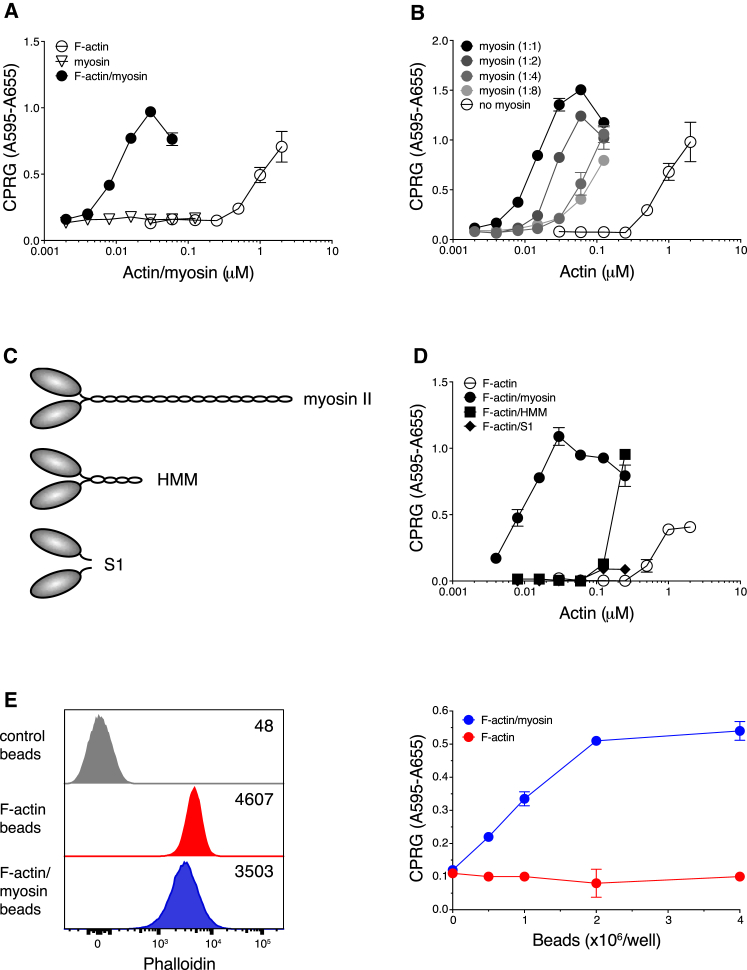
Myosin II Potentiation of F-Actin Agonistic Activity Requires an Intact Myosin Heavy-Chain Tail (A, B, D, and E) Titration of pre-polymerized stimuli on B3Z-mDNGR-1-Syk reporter cells. Graphs show absorbance after addition of β-galactosidase substrate to lysed cells. Plotted data represent mean ± SD of duplicate wells. (A) Comparison of *in vitro* polymerized F-actin (open circles), myosin II (open triangles), and an equimolar mixture of F-actin and myosin II (filled circles). (B) F-actin alone (open circles) and F-actin mixed with myosin II at various molar ratios as indicated. (C) Schematic representation of the heavy chains of myosin II (top) and its proteolytic cleavage products heavy meromyosin (HMM, center) and monomeric S1 fragments (bottom). For clarity, the essential and regulatory light chains have been omitted. (D) F-actin alone (open circles) and an equimolar mixture of F-actin with intact myosin II (filled circles), HMM (filled squares), and myosin II S1 fragments (filled diamonds). (E) Flow cytometry and functional analysis of beads coated with phalloidin-stabilized F-actin. Left panel: overlay histogram for uncoated beads (gray) labeled with an equal amount of Alexa 488-labeled phalloidin and beads coated with Alexa 488-phalloidin-stabilized F-actin only (red) or additionally coated with myosin II (blue). Numbers in the histogram overlay represent mean Alexa 488-phalloidin fluorescence intensity. Right panel: titration of beads coated with F-actin alone (red circles) or F-actin and myosin II (blue circles) on B3Z-mDNGR-1-Syk reporter cells. Data represent 7 (A), 2 (B), and 4 (D and E) experiments with similar results.

To examine the functional significance of the binding assay results, we examined the ability of F-actin ± myosin II to stimulate reporter cells in which DNGR-1 signaling via Syk is measured by activation of an NFAT reporter (Sancho et al., 2009). As shown previously (Ahrens et al., 2012), F-actin alone stimulated reporter activity but only did so at concentrations equal to or above 1 μM (Figure 2A). Addition of F-actin pre-mixed with myosin II resulted in a 2-log leftward shift of the dose-response curve (Figure 2A). We observed a significant shift in the dose-response curve even when we decreased the amount of myosin II to a molar ratio of 1:8 relative to actin (Figure 2B). As expected, myosin II by itself had no stimulatory activity (Figure 2A).

We next compared intact myosin II with its cleavage products, heavy meromyosin (HMM) and S1 fragments (Figure 2C). We found that HMM in complex with F-actin was less effective at inducing DNGR-1 signaling than intact myosin but was still superior to S1 fragments, which were largely inactive (Figure 2D). These data suggest that the F-actin bundling activity of myosin II, which is lost in S1 fragments and partially retained in HMM, is required for its ability to potentiate DNGR-1 recognition (see Discussion). Finally, we created a particulate DNGR-1 stimulus by coupling biotinylated F-actin and myosin II to streptavidin (SA)-coated polystyrene beads. To normalize for the amount of F-actin deposited onto beads, monomeric actin was polymerized in the presence of phalloidin, which stabilizes F-actin but does not interfere with the binding of DNGR-1 (Ahrens et al., 2012). Addition of myosin II to phalloidin-stabilized, bead-bound F-actin did not greatly affect the phalloidin signal (Figure 2E, left panel), confirming that equal amounts of F-actin were displayed on both F-actin and F-actin and myosin II bead preparations. However, when tested in a reporter assay, beads coupled to F-actin and myosin II triggered signaling in a dose-dependent manner, whereas beads coupled to F-actin alone were largely inactive (Figure 2E, right panel), presumably because the amount of F-actin coupled to beads was below the concentration required for reporter cell triggering (Figure 2A). These data suggest that myosin II does not simply act to stabilize F-actin but bundles F-actin filaments in a manner that favors DNGR-1 binding. Consistent with that notion, high doses of myosin on the beads can start to inhibit DNGR-1 signaling, even though they do not decrease F-actin content, perhaps because of overbundling (data not shown). We conclude that F-actin and myosin II complexes constitute a superior DNGR-1 ligand, at least partly because of myosin-dependent cross-linking of actin filaments.

### Antigen-F-Actin and Myosin II Beads Are Taken up and Cross-Presented by cDC1s in a DNGR-1-Dependent Manner

**Figure 3:**
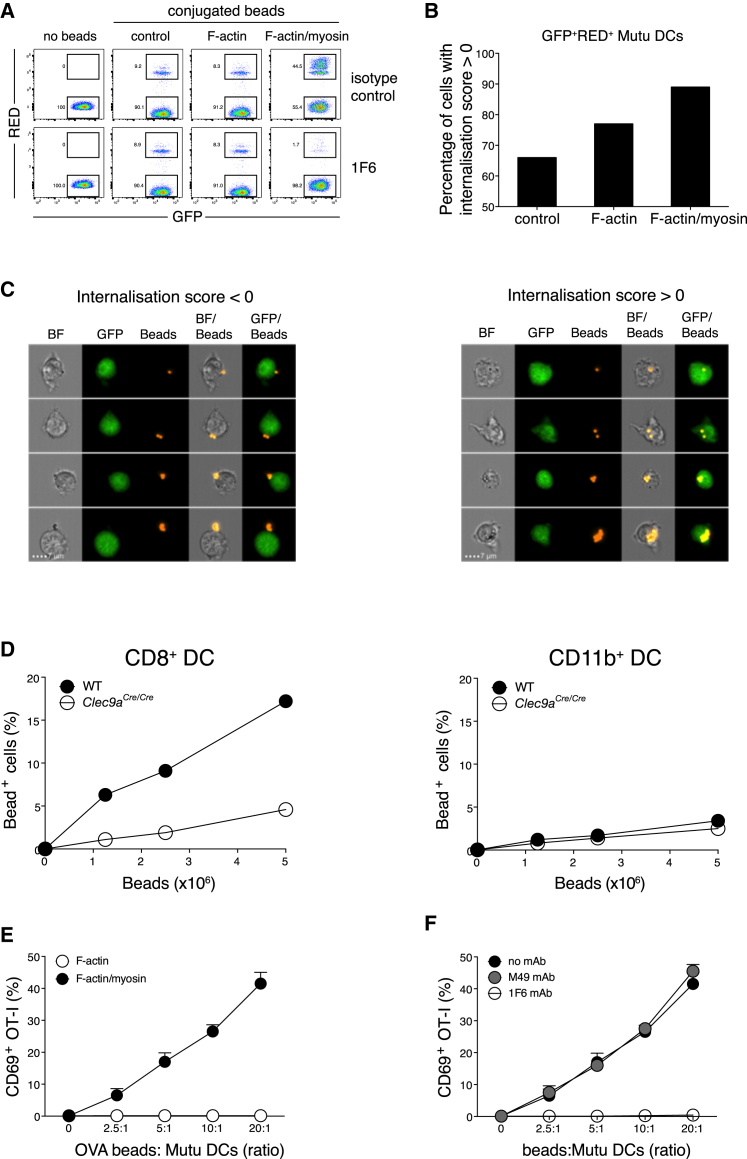
cDC1s Phagocytose and Cross-Present F-Actin and Myosin II-Coated Beads in a DNGR-1-Dependent Manner (A–D) Association of red fluorescent beads with DCs was analyzed by flow cytometry. (A) Co-culture of MutuDCs with red fluorescent beads coated with either F-actin or F-actin and myosin II or mock-treated in the presence of isotype control antibody (upper panels) or 1F6 antibody (lower panels). Rectangular gates show the percentage of MutuDCs that either contained internalized or surface-bound beads (double-positive, i.e., GFP^+^Red^+^ cells) or was devoid of beads (single positive, i.e., GFP^+^ cells). (B) ImageStream analysis of MutuDCs after incubation with F-actin, F-actin and myosin II, or control beads. The histogram shows the percentage of double-positive MutuDCs with an internalization score > 0. (C) Image gallery of individual MutuDCs, after incubation with F-actin and myosin II beads that are representative of cells with an internalization score < 0 (left panel), and those with an internalization score > 0 (right panel) indicative of true uptake. Scale bars shown in the lower panels correspond to a size of 7 μm. (D) Association of splenic CD8α^+^ cDC1s (left panel) and CD11b^+^ cDC2s (right panel) with F-actin and myosin II beads. (E and F) CD69 expression on OT-I T cells after overnight culture with MutuDCs in the presence of beads coated with OVA and additionally coupled to F-actin and myosin II (filled circles) or F-actin (open circles) (E) or F-actin and myosin II and OVA-coated beads in the absence (black filled circles) or presence (gray filled circles) of isotype-matched irrelevant specificity control or 1F6 antibody (open circles) (F). Plotted data in (E) and (F) represent mean ± SD of duplicate wells. Data are representative of 3 (A and E) and 2 (B, C, D, and F) independent experiments.

To test the interaction of F-actin and myosin II with DNGR-1 on cDC1s, we added F-actin and myosin II-decorated red fluorescent beads to GFP-expressing MutuDC1940 cells—an immortalized splenic mouse cDC1 line (Fuertes Marraco et al., 2012), henceforth referred to as MutuDCs—and analyzed bead association with the cells by flow cytometry. Whereas only a small percentage of MutuDCs associated with uncoated control beads or F-actin-coated beads, up to 50% of MutuDCs associated with F-actin and myosin II beads (Figure 3A). Furthermore, whereas most MutuDCs associated with a single bead in the group of control or F-actin beads, incubation of MutuDCs with F-actin and myosin II beads resulted in many DCs associated with multiple beads, leading to a characteristic laddering pattern (Figure 3A, top row, right). Multispectral imaging flow cytometry (ImageStream) established that fluorescence-activated cell sorting (FACS)-based analysis of cell and bead association correlated with actual bead uptake: in the case of control or F-actin beads, at least two-thirds of double-positive MutuDCs had internalized particles, whereas this number rose to almost 90% for F-actin and myosin II beads (Figure 3B). To validate the internalization algorithm, image galleries of randomly selected cells were examined. Visual inspection of individual bead-positive MutuDCs from the F-actin and myosin II bead sample showed that green and red fluorescence signals co-localized in cells with an internalization score > 0 (Figure 3C, right panel), whereas they remained largely separate in cells with an internalization score < 0 (Figure 3C, left panel). Internalization of F-actin and myosin II beads, but not control or F-actin beads, could be inhibited by pre-treatment of the MutuDCs with a monoclonal antibody (mAb) specific for DNGR-1 (Figure 3A, bottom row), demonstrating that uptake was DNGR-1 dependent. The mAb did not inhibit uptake of other particles by MutuDCs, including bacteria or zymosan (data not shown). Using DC-enriched splenocyte fractions from wild-type (WT) and DNGR-1-deficient mice, we confirmed that F-actin and myosin II beads were preferentially taken up by primary CD8α^+^ cDC1s in a DNGR-1-dependent manner (Figure 3D, left). Consistent with CD11b^+^ conventional type 2 DCs (cDC2s) not expressing DNGR-1, we observed only a small amount of receptor-independent uptake (Figure 3D, right), similar to that seen with DNGR-1-deficient cDC1 (Figure 3D, left).

To assess the effect of F-actin and myosin II on cross-presentation, we fed F-actin and myosin II beads that were additionally coated with ovalbumin (OVA) to MutuDCs and incubated the latter with naive OT-I T cells. At these coating concentrations, OVA coupled to F-actin beads was not detectably cross-presented, as determined by the lack of upregulation of CD69 on OT-I T cells (Figure 3E). In contrast, OT-I T cells were activated by MutuDCs incubated with increasing doses of OVA and F-actin and myosin II beads (Figure 3E). This could be blocked by anti-DNGR-1 mAb, but not by an isotype-matched control mAb (Figure 3F). We conclude that beads bearing F-actin and myosin II complexes are efficiently phagocytosed by cDC1 and lead to cross-presentation of bead-bound antigen in a DNGR-1-dependent manner.

### Immunization with Antigen-F-Actin and Myosin II Beads Generates an Effective CTL Response

**Figure 4:**
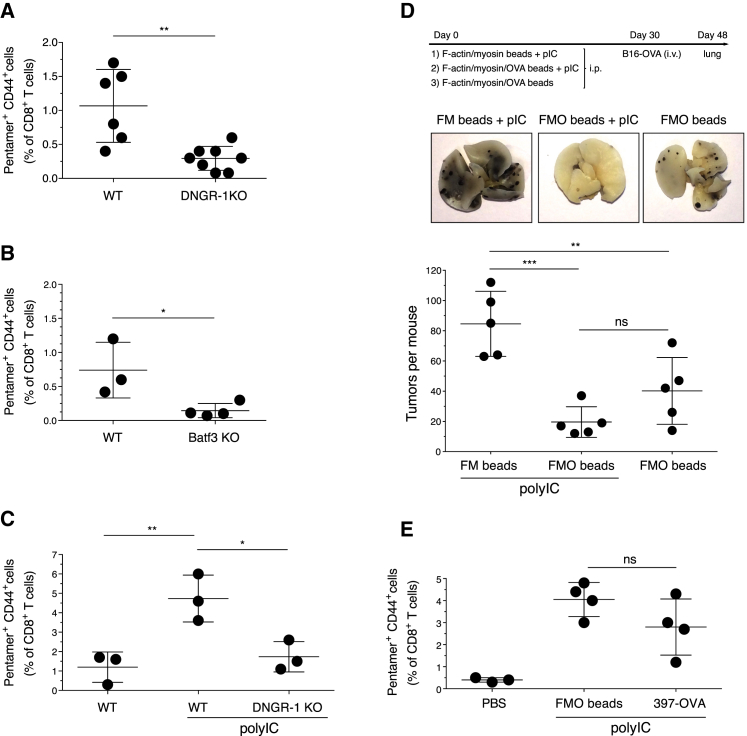
F-Actin and Myosin II Beads Are an Efficient Antigen Delivery Platform for DNGR-1-Dependent Induction of CTL Responses *In Vivo* Induction of OVA-specific CD8 T cell responses following immunization with F-actin and myosin II/OVA (FMO) beads. (A, B, C, and E) Percentage of H-2K^b^/SIINFEKL pentamer CD44 double-positive cells of total CD8^+^ T cells. (A and B) Comparison of WT and DNGR-1-deficient mice (A) or WT and Batf3 KO mice (B). (C) Same as (A), but FMO beads were mixed or not mixed with poly I:C (25 μg/mouse). (D) B16-OVA tumor rechallenge of mice immunized with FMO beads ± poly I:C or F-actin and myosin II (FM) beads + poly I:C as indicated. Shown are the experimental setup (top), images of one representative lung from each treatment group (center), and number of lung tumors per mouse (bottom). (E) Mice were injected i.p. with PBS vehicle control or with 4 × 10^7^ FMO beads or 2 μg 397-OVA antibody, both mixed with poly I:C (25 μg/mouse). Data in (A) were compiled by pooling mice from different experiments. Otherwise, data are representative of 2 (B and E) and 3 (C and D) experiments with similar results. NS, not significant. ^∗^p < 0.05, ^∗∗^p < 0.01, ^∗∗∗^p < 0.001. Plotted data depict mean ± SD with each dot representing an individual mouse.

The preceding results suggested that F-actin and myosin II could be used to target antigen to cross-presenting DNGR-1^+^ cDC1 *in vivo* and generate CTL responses. Systemic injection of F-actin and myosin II and OVA-coated beads, but not beads coated solely with F-actin and OVA (data not shown), was sufficient to lead to expansion of endogenous OVA-specific CD8^+^ T cells (Figure 4A). The response was greatly reduced in mice lacking DNGR-1 (Figure 4A) or in Batf3-deficient mice lacking cDC1 (Figure 4B). As might be expected, combining F-actin and myosin II and OVA beads with an innate immune stimulus such as polyinosinic:polycytidylic acid (poly I:C) that strongly activates cDC1 (Lauterbach et al., 2010, Longhi et al., 2009, Schulz et al., 2005) augmented T cell priming, which remained dependent on DNGR-1 (Figure 4C). Next, we immunized mice and rested them for 4 weeks to allow development of T cell memory, which was then tested by challenge with B16-OVA melanoma cells and assessment of tumor burden (Figure 4D, top). The lungs of mice immunized with OVA-coupled F-actin and myosin II beads were almost tumor-free 18 days after challenge (Figure 4D); there was a trend (albeit without reaching statistical significance) for tumor burden to be even lower when the same beads were administered with poly I:C (Figure 4D). In contrast, control mice that had been immunized with F-actin and myosin II beads + poly I:C (without OVA) had a high number of lung tumors (Figure 4D), indicating that the anti-tumor response was OVA specific.

Antigens coupled to mAbs against DNGR-1 have been used to target cDC1 *in vivo* and elicit potent CTL responses, especially when given with poly I:C (Caminschi et al., 2008, Idoyaga et al., 2011, Sancho et al., 2008). To directly compare antibody- versus ligand-mediated targeting of antigen to DNGR-1, we either injected F-actin and myosin II and OVA beads or an anti-DNGR-1-OVA fusion antibody and assessed the generation of OVA-specific CTL response 8 days later. The amount of OVA in the bead inoculum (300–500 ng per mouse) (data not shown) was comparable to that contained in the antibody-OVA fusion protein (600 ng per mouse) (data not shown), yet both reagents induced a comparable OVA-specific CD8 T cell response (Figure 4E). We conclude that F-actin and myosin II beads are at least as efficient as anti-DNGR-1 antibodies in delivering antigen to cross-presenting cDC1s *in vivo*.

### Myosin II Is Required for Dead Cells to Stimulate DNGR-1-Dependent Responses

**Figure 5:**
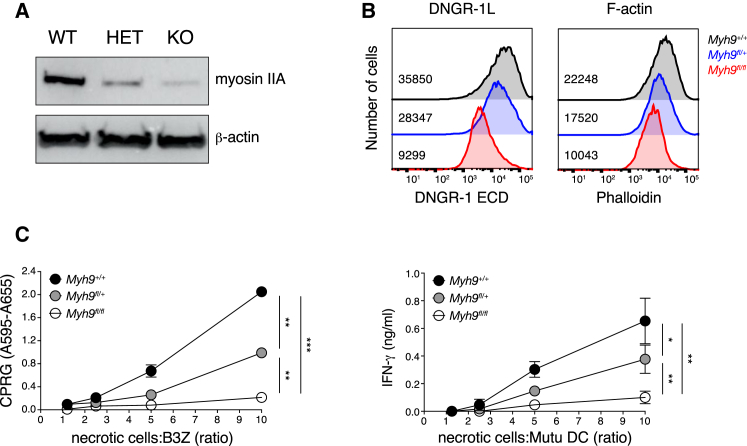
Necrotic Cells Lacking Myosin II Are Impaired in Their Ability to Stimulate DNGR-1 Signaling and to Serve as Substrates for Cross-Presentation by cDC1 (A and B) Western blot (A) and flow cytometry (B) analysis of CD23^+^ cells from CD23^Cre^ x Myh9^fl/fl^ (KO), CD23^Cre^ x Myh9^fl/+^ (heterozygous [HET]), and CD23^Cre^ x Myh9^+/+^ (WT) mice. (A) Expression of myosin IIA (upper panel) and β-actin (lower panel). (B) Overlay histograms of necrotic cells stained with DNGR-1 ECD (left panel) and phalloidin (right panel). Numbers inside graphs represent mean fluorescence intensity for each of the three samples. (C) Necrotic cells were added at various doses as indicated to B3Z-mDNGR-1-Syk reporter cells (left panel) or MutuDC and pre-activated OT-I co-cultures (right panel). Graphs show absorbance after addition of β-galactosidase substrate to lysed cells (left panel) or concentration of IFN-γ in the supernatant (SN) of overnight cultures (right panel). Plotted data represent mean ± SD of duplicate wells. Data are representative of 2 (A and B), 4 (C, left panel), and 3 (C, right panel) independent experiments. ^∗^p < 0.05, ^∗∗^p < 0.01, ^∗∗∗^p < 0.001.

Finally, to explore the significance of myosin II in the context of physiological DNGR-1-mediated recognition, we tested dead cells lacking myosin IIA as substrates for cross-presentation by cDC1. We used dead B cells for these experiments because B cells express myosin IIA (encoded by the *Myh9* gene), but not the other two myosin isoforms, namely, myosin IIB and myosin IIC; therefore, *Myh9*-deficient B cells are overall deficient in myosin II (Hoogeboom et al., 2018). Western blot analysis of cell lysates confirmed the absence of myosin IIA in CD23^+^ B cells from CD23^Cre^ x Myh9^fl/fl^ mice (Figure 5A). The faint band in the *Myh9*-deficient cells is most likely due to a small contamination of the CD23-enriched cells with *Myh9*-sufficient cells (Hoogeboom et al., 2018). Total actin content was similar (Figure 5A), although myosin IIA-deficient cells showed a 2-fold decrease in phalloidin staining (Figure 5B, right panel), suggesting that the actin cytoskeleton in cells lacking myosin IIA is skewed toward G-actin. Disproportionate to the slight reduction in phalloidin staining, myosin IIA-deficient B cells displayed a marked decrease in staining with DNGR-1 ECD compared to myosin IIA-sufficient controls (Figure 5B, left panel). Similarly, while necrotic myosin IIA-sufficient B lymphocytes efficiently stimulated DNGR-1 reporter cells (Figure 5C, left panel), the responses were almost abolished when necrotic, myosin IIA-deficient B lymphocytes were used as stimulator cells (Figure 5C, left panel). B lymphocytes containing one allele of *Myh9* showed an intermediate phenotype (Figures 5B and 5C, left panel), suggesting a gene dosage effect that matches our earlier observation of a dose-dependent effect of myosin II on DNGR-1 ECD binding to *in vitro* polymerized F-actin (Figure 2B). Finally, we coated necrotic B cells with OVA and assessed their ability to serve as substrates for cross-presentation by cDC1 based on the interferon γ (IFN-γ) accumulation in co-cultures with MutuDC/OT-I T cells. Again, myosin IIA-deficient necrotic B lymphocytes were poor antigen donors compared to WT control cells, and *Myh9* heterozygous B cells displayed an intermediate phenotype (Figure 5C, right panel). IFN-γ accumulation was not seen in cultures in which MutuDCs were omitted, excluding direct presentation by the dead B cells (data not shown). We conclude that myosin II is required in dead cells to facilitate F-actin recognition by DNGR-1 and the ensuing cross-presentation of dead cell-associated antigens.

## Discussion

Cross-presentation of dead cell-associated antigens plays a key role in self-tolerance, as well as in the priming of immune responses to tumors and some viruses (Heath and Carbone, 2001, Joffre et al., 2012). cDC1s have emerged as central players in cross-presentation *in vivo* and possess specialized receptors, such as DNGR-1, that facilitate the extraction of antigens from internalized cell corpses and the shuttling of those antigens into an major histocompatibility complex class I (MHC class I) presentation pathway. DNGR-1 binds to exposed actin filaments on cell corpses, but such filaments are rarely naked, raising the possibility that ABPs might interfere with or, conversely, potentiate DNGR-1 recognition. Here, we show that myosin II acts in the latter fashion, greatly increasing DNGR-1 binding to F-actin and subsequent cross-presentation by cDC1. Notably, myosin II appears to be an integral component of the physiological trigger for DNGR-1, because its loss from antigen donor cells decreases receptor binding, signaling, and cross-presentation of dead cell-associated antigens. These findings shed light on the mechanisms used by the innate immune system to detect cell death and can be exploited to generate an optimized DNGR-1 ligand that can be used to target cDC1.

A report suggests that DNGR-1 binding to F-actin requires accessory proteins with a calponin-homology domain (Zhang et al., 2012), which is found in some ABPs (Dominguez, 2004). In our hands, calponin-homology domain ABPs such as actinin or spectrin did not potentiate DNGR-1 binding. However, the same was not true of myosin II, which increased binding by at least 50-fold. Myosin II did not merely increase the stability of F-actin, because the same amount of F-actin in solution or coupled to beads was much less effective in functionally engaging DNGR-1. What is it then that makes myosin II so special? Myosin II heavy chain has an elongated rod-like tail, which forms an α-helical coiled-coil that causes dimerization (Figure 2C) and allows myosin dimers to organize into filaments. The ability to potentiate F-actin agonistic activity was only partially retained in HMM, which has a shorter tail but is still dimeric, and was almost completely lost in S1 fragments, which lack the tail and are monomeric. These data suggest that the α-helical region of the heavy chain, which is responsible for creating the coiled-coil tail of the myosin heavy-chain dimer, is a critical determinant for creating, with F-actin, an enhanced DNGR-1 agonist. Myosin II-mediated cross-linking could lead to a spatial patterning of F-actin filaments that favors the binding of the “free” CTLD of the DNGR-1 homodimer to an adjacent filament (*trans* binding). Indeed, we found that increased binding to F-actin and myosin II requires DNGR-1 to be dimeric, arguing that myosin does not so much increase the affinity of F-actin for the DNGR-1 CTLD per se but, rather, affects the interaction indirectly by promoting the binding co-operativity of the two CTLDs.

Beads coupled to F-actin and myosin II were internalized by cDC1 in a DNGR-1-dependent manner, consistent with studies showing that the receptor recycles through the endocytic pathway and can capture cargo (e.g., anti-DNGR-1 antibody) at the cell surface for intracellular delivery (Caminschi et al., 2008, Sancho et al., 2008). Co-coupling of OVA to F-actin and myosin II beads created a powerful immunogen for generating OVA-specific CTL responses *in vivo* when combined with adjuvants such as poly I:C. Basal immunogenicity was also seen when beads were administered without adjuvant, although this might be attributable to traces of endotoxin or other microbial contamination in commercial actin and myosin II preparations (data not shown). Importantly, side-by-side comparison established that antigen delivery by F-actin and myosin II beads is as potent as antibody-mediated delivery in targeting DNGR-1 and inducing a CD8^+^ T cell response. CD8^+^ T cell responses are proving very beneficial in cancer patients treated with checkpoint blockade agents, and it has been suggested that response rates could be improved by vaccination (Baumeister et al., 2016, Ott et al., 2017). We propose that beads coupled to F-actin and myosin II, in addition to proving useful for probing DNGR-1 function, may therefore provide an alternative strategy to antibodies for targeting antigens to cross-presenting cDC1 (Caminschi et al., 2012), which could be exploited for vaccination in cancer and other settings.

## Experimental Procedures

### Mice

C57BL/6 mice were purchased from Charles River (Margate, UK). Clec9a^cre/cre^, Batf3^−/−^ (a gift from Ken Murphy, Washington University, St. Louis), OT-I x Rag1^−/−^, and CD23^Cre^ x Myh9^fl/fl^ mice were bred at the animal facility of the Francis Crick Institute. Mice were used from 6 to 12 weeks of age. For *in vivo* experiments, mice were gender matched, and littermates of the same sex were randomly assigned to treatment or control groups. Animal experiments were performed in accordance with national and institutional guidelines for animal care and were approved by the Francis Crick Institute Biological Resources Facility Strategic Oversight Committee (incorporating the Animal Welfare and Ethical Review Body) and by the Home Office, UK.

### Reagents

Purified rabbit muscle actin, α-actinin, myosin II, tropomyosin and troponin, and Alexa 488-labeled phalloidin were obtained from Cytoskeleton. Spectrin was from Sigma. 1 μm red fluorescent (580/605) and 2 μm non-fluorescent SA-coated microbeads were from Life Technologies and Polysciences, respectively. FLAG-tagged recombinant dimeric mouse DC, NK lectin group receptor-1 (mDNGR-1) ECD and monomeric mDNGR-1 CTLD were prepared as described (Ahrens et al., 2012). Horseradish peroxidase (HRP)-conjugated mouse anti-FLAG (M2) antibody (Sigma) and phycoerythrin (PE)-conjugated rat-anti-DNGR-1 antibody (1F6) were was used for DNGR-1 detection by dot blot and flow cytometry, respectively. Rabbit myosin IIA antibody and HRP-conjugated rabbit β-actin antibody were from Cell Signaling Technology. HRP-conjugated goat-anti-rabbit antibody was from Southern Biotech. OVA (Calbiochem) was conjugated to biotin using a kit (Life Technologies) as per the manufacturer’s instructions. R-PE-conjugated H-2K^b^/SIINFEKL pentamer was from Proimmune. Fluorophore-labeled monoclonal antibodies against CD69, CD3, CD8, CD44, CD19, CD11c, and CD11b were from BD Biosciences. Poly I:C was from InvivoGen.

For generation of recombinant 397/OVA fusion protein, the variable regions of the heavy and the light chains of the DNGR-1-specific antibody 397 (Sancho et al., 2008) were identified by 5′ RACE (rapid amplification of cDNA ends). These 397-specific variable regions were cloned into a pVITRO-based expression plasmid containing a rat immunoglobulin 2b (IgG2b) constant region fused to the model antigen OVA at the C terminus of the antibody (Dodev et al., 2014). Recombinant 397/OVA fusion antibody was expressed by transient transfection in HEK293T cells and purified from cell supernatants using protein G columns.

### Cells

B16-OVA melanoma cells and B3Z cells stably transduced with mouse *Clec9a* and *Syk* (B3Z-mDNGR-1-Syk cells) were grown in RPMI 1640 containing 10% fetal calf serum (FCS), 2 mM glutamine, 50 μM 2-mercaptoethanol, 100 units/mL penicillin, and 100 μg/mL streptomycin (R10). B3Z cells express a β-galactosidase (β-gal) reporter for NFAT (Sancho et al., 2009). The MutuDC1940 line was a gift from Hans Acha-Orbea (Lausanne, Switzerland) and was cultured in Iscove’s Modified Dulbecco’s Medium (IMDM) medium containing 10% FCS, 50 μM 2-mercaptoethanol, 100 units/mL penicillin, and 100 μg/mL streptomycin. All media and media supplements were from Life Technologies except for FCS (Source Bioscience). For experiments involving myosin-deficient primary cells, pooled spleen and lymph node (LN) cells from CD23^Cre^ x Myh9^fl/fl^ and control mice were enriched for CD23^+^ B lymphocytes by positive selection using magnetic beads against mouse CD23 (Miltenyi). Typical purity was at least 85%. Enriched cells were either lysed directly for western blot (WB) analysis or incubated with OVA (10 mg/mL) for 1 hr and made necrotic by one freeze-thaw round.

### Dot Blot Binding Assay

Binding of DNGR-1 to *in vitro* polymerized F-actin, F-actin and myosin II complexes, or HeLa cell lysate was analyzed by dot blot as described previously (Ahrens et al., 2012). F-actin, F-actin and myosin II complexes, or cell lysate was transferred onto nitrocellulose membranes by gravity flow using a dot blot apparatus. Post-transfer, nitrocellulose (NC) membranes were blocked, cut into strips, and either probed directly as per the published protocol or incubated with the purified ABPs in blocking solution for 1–2 hr, washed, and then probed.

### Preparation of Soluble F-Actin and Myosin II

F-actin was prepared as described (Ahrens et al., 2012). Briefly, G-actin (10 mg/mL, 200 μM) stock was diluted 1:10 into F-actin buffer and left at room temperature (RT) for at least 1 hr to induce filament formation. F-actin (20 μM) was then diluted 1:4 in PBS, and myosin II (stock at 20 μM or 10 mg/mL) was added at equimolar concentration. The mixture (F-actin and myosin at 4 μM) was incubated for 1 hr at RT and adjusted to the final assay concentration (top dose) with PBS. Dilution series of F-actin and myosin II preparations were prepared in PBS and used directly for dot blot and reporter cell assays.

### Coupling of F-Actin, Myosin II, and OVA to Microspheres

Biotinylated F-actin was prepared by mixing equal amounts (20 μL) of G-actin and biotinylated G-actin (both at 20 μM or 1 mg/mL), followed by addition of 5 μL of G-actin buffer and 5 μL of 10× F-actin buffer to start the polymerization reaction (1 hr at RT). 12.5 μL of biotinylated F-actin (16 μM) was mixed with 27.5 μL of PBS and 10 μL of myosin II (20 μM or 10 mg/mL) for a final concentration of 4 μM each and incubated for 1 hr at RT. Biotin-F-actin and myosin II was diluted 1:4 with PBS and, 100 μL was added to 20 μL of SA-coated beads (2 μm; Polysciences), which had been washed twice with wash buffer (PBS + 1% BSA), for 30 min on ice. After washing, F-actin and myosin beads were resuspended with 100 μL of biotinylated OVA (0.4 mg/mL) and incubated for a further 30 min on ice. Washed beads were resuspended in wash buffer and sonicated (2 × 1 min) in a water bath sonicator before storage. F-actin and myosin and OVA beads were tested for endotoxin using a chromogenic Limulus Amebocyte Lysate assay (Thermo Scientific) and found to contain ≤0.15 endotoxin units (EUs)/10^6^ beads.

For uptake assays, non-fluorescent microspheres were replaced with fluorescent SA-coated microspheres, and the addition of biotinylated OVA was omitted.

For comparison of F-actin and F-actin and myosin II beads with a normalized amount of F-actin, a mixture of biotinylated G-actin and unlabeled G-actin was polymerized in the presence of an equimolar concentration (20 μM) of Alexa 488-labeled phalloidin. Phalloidin-stabilized biotinylated F-actin (1 μM) was coupled to 8 × 10^7^ SA-coated microspheres, washed several times, split into two samples, and either left untreated or incubated with myosin II (25–50 μg).

### Phagocytosis Assay

5 × 10^5^–10 × 10^5^ MutuDCs or CD11c-enriched splenocytes were incubated with coated (F-actin or F-actin and myosin) or uncoated (control) red fluorescent beads in a total volume of 1 mL in 24-well flat bottom plates for 2 hr at 37°C. To facilitate bead uptake, plates were centrifuged at 1,000 rpm for 2 min at the start of incubation. Cultures were washed and analyzed either directly (MutuDCs) by flow cytometry or, in the case of splenic DCs, after staining for surface markers (CD11b, CD8α, and CD11c) to distinguish splenic DC subsets. To verify and quantitate bead uptake accurately, double-positive (GFP^+^RED^+^) MutuDCs were acquired on a multispectral imaging flow cytometer (ImageStream mkII; Amnis, Seattle, WA). True uptake of fluorescent beads by MutuDCs was determined using an internalization feature that looks at the degree to which RED fluorescent beads co-localize with MutuDCs defined by tight masking on the GFP signal. Confirmation of internalization was assessed by examination of the image gallery of GFP^+^RED^+^ MutuDCs. At least 10,000 single and focused cells were collected per sample, and data were analyzed using IDEAS 3.0.245 software (Amnis, Seattle, WA).

### *In Vitro* Cross-Presentation

F-actin and OVA- or F-actin and myosin and OVA-coated beads or necrotic B cells were added to MutuDCs (1 × 10^5^/well) at the indicated ratio and cultured in 96-well round-bottom plates at 37°C. To facilitate bead or dead cell uptake, plates were centrifuged at 1,000 rpm for 2 min at the start of the incubation. Meanwhile, naive T cells were isolated from pooled lymph nodes of OT-I x Rag1 knockout (KO) mice and depleted of antigen presenting cells (APCs) as described (Sancho et al., 2009). APC-depleted OT-I T cells (5 × 10^4^/well) were added to the Mutu cultures 4 hr after culture initiation. After overnight incubation, cells were washed, stained with anti-CD3 and anti-CD69 mAbs, and analyzed by flow cytometry.

In experiments involving necrotic B cells, naive OT-I T cells were replaced with pre-activated OT-I T cells that were generated as described previously (Hanč et al., 2016b). Briefly, pooled, red blood cell-depleted spleen and lymph node cells from OT-I x Rag1 KO mice were cultured for 5 days with SIINFEKL peptide (0.1–1 nM) and mouse interleukin-2 (mIL-2; 25 U/mL), which was added on day 3. Pre-activated OT-I T cells (5 × 10^4^/well) were then added to 4 hr Mutu and dead cell co-cultures, and OT-I T cell activation was determined by measuring IFN-γ in the supernatant of overnight cultures by ELISA.

### *In Vivo* Immunization with F-Actin and Myosin II Beads

Mice were injected intraperitoneally (i.p.) or intravenously (i.v.) with 4 × 10^7^ F-actin and myosin/OVA beads or control beads in PBS in a total volume of 0.2 mL. In some experiments, beads were mixed with poly I:C (25 μg/mouse) before injection. 6–8 days later, red blood cell-lysed splenocyte suspensions were prepared from spleens of injected mice and stained with H-2K^b^/SIINFEKL pentamer reagent, followed by anti-CD8α, anti-CD44, and anti-CD19 antibodies. Pentamer-positive, i.e., OVA-specific, CD8^+^ T cells were analyzed by flow cytometry. For tumor rechallenge experiments, mice were injected with beads i.p. and rested for at least 28 days. All mice were then injected i.v. with 2 × 10^5^ B16-OVA cells; after 18 days, the number of tumor foci was counted in lungs from injected mice.

### Flow Cytometry

Samples were counterstained with DAPI to exclude dead cells and acquired on an LSRFortessa (BD Biosciences). Data were analyzed using FlowJo software (Tree Star).

### NFAT Reporter Assay in B3Z Cells

For measuring the agonistic activity of F-actin and myosin-modified F-actin, we used an NFAT reporter assay as described previously (Sancho et al., 2009). B3Z-mDNGR-1-Syk cells were plated in 96 well plates (1 × 10^5^ cells/well) in the presence of added stimuli as indicated. Stimulation of reporter cells was performed in R10 medium except for the experiment in Figure 2E, which was carried out in serum-free medium (AIM-V; Life Technologies). After overnight culture, cells were washed once in PBS and LacZ activity was measured by lysing cells in chlorophenol red-β-D galactopyranoside (CPRG) (Roche)-containing buffer. 1–4 hr later, optical density 595 (OD_595_) was measured using optical density 655 (OD_655_) as a reference.

### Statistics

Statistical analysis of *in vivo* immunization experiments was performed using one-way ANOVA. *In vitro* experiments comparing myosin IIA-sufficient and myosin IIA-deficient B cells were analyzed by two-way ANOVA.
